# Black and Hispanic Men Perceived to Be Large Are at Increased Risk for Police Frisk, Search, and Force

**DOI:** 10.1371/journal.pone.0147158

**Published:** 2016-01-19

**Authors:** Adrienne N. Milner, Brandon J. George, David B. Allison

**Affiliations:** 1 Department of Sociology, University of Alabama at Birmingham, Birmingham, AL 35294, United States of America; 2 Nutrician Obesity Research Center, University of Alabama at Birmingham, Birmingham, AL 35294, United States of America; 3 Minority Health and Health Disparities Research Center, University of Alabama at Birmingham, Birmingham, AL 35294, United States of America; 4 Center for the Study of Community Health, University of Alabama at Birmingham, Birmingham, AL 35294, United States of America; 5 School of Public Health, University of Alabama at Birmingham, Birmingham, AL 35294, United States of America; 6 Office of Energetics, University of Alabama at Birmingham, Birmingham, AL 35294, United States of America; Johns Hopkins Bloomberg School of Public Health, UNITED STATES

## Abstract

Social justice issues remain some of the most pressing problems in the United States. One aspect of social justice involves the differential treatment of demographic groups in the criminal justice system. While data consistently show that Blacks and Hispanics are often treated differently than Whites, one understudied aspect of these disparities is how police officers' assessments of suspects' size affects their decisions. Using over 3 million cases from the New York Police Department (NYPD) Stop, Question, and Frisk (SQF) Database, 2006–2013, this study is the first to explore suspects' race, perceived size, and police treatment. Results indicate that tall and heavy black and Hispanic men are at the greatest risk for frisk or search. Tall and heavy suspects are at increased risk for experiencing police force, with black and Hispanic men being more likely to experience force than white men across size categories.

## Introduction

According to the New York City medical examiner, on July 17^th^, 2014, Eric Garner, an unarmed black man, died of a heart attack caused by Staten Island police pinning him to the ground and applying a chokehold [1]. This case, along with the fatal shooting of unarmed black teenager, Michael Brown, in Ferguson MO on August 9^th^, 2014, has drawn public awareness to issues surrounding racial profiling and excessive use of police force. There have been several other recent cases in which unarmed black men have been killed by the police [2,3] including: Dante Parker, who died after being stunned by a Taser gun multiple times by police in Victorville, CA on August 12^th^, 2014; Walter Scott, who video footage [4] shows was shot multiple times while running from an officer in North Charleston, SC on April 4th, 2015; and Freddie Gray, who suffered a fatal spine injury after being apprehended by Baltimore police on April 12^th^, 2015; there are also many historic cases (e.g., Abner Louima, Amadou Diallo, Patrick Dorismond [5]). A recent report [6] shows that black males are 21 times more likely than their white counterparts to be shot and killed at the hands of police at a rate of 31.17 deaths per million as compared to 1.47 deaths per million for white males. While the data clearly demonstrate racial disparities, an understudied aspect surrounding race and the use of force is how officers’ perceptions of suspects’ size may affect police treatment. Aside from being black, Garner (6’3, 350 lbs.), Brown (6’4, 292 lbs.), and Parker (5’10, 274 lbs.), were physically large men at the time of their deaths [1,7,8], and this may, along with their race, have affected the perceptions and actions of police officers. Human visual perception is not an objective or unbiased process [9]; an officer's perception of the size of a suspect—and thus of the potential physical danger posed by that suspect—may be influenced by the suspect's race, independent of actual size. Size, like beauty, may be in the eyes of the beholder.

In fact, when describing his encounter with Michael Brown to the grand jury, Officer Darren Wilson testified that he “felt like a five-year-old holding onto Hulk Hogan. that's just how big [Brown] felt and how small [Wilson] felt.”[10] Wilson also testified that “[Brown] had the most aggressive face. That’s the only way [Wilson] can describe it, it looks like a demon, that’s how angry [Brown] looked.” Wilson explained that he fired 12 shots at Brown because “at this point it looked like [Brown] was almost bulking up to run through the shots, like it was making him mad that [Wilson was] shooting at [Brown] [11].” Not only does Wilson’s description of the events indicate that Brown’s size influenced Wilson’s actions, his claims that Brown became stronger after being hit by bullets, referring to Brown as “it,” and comparing Brown to an aggressive demon also speaks to racial issues.

Negative images of black men in American society have evolved from slavery to modern times and continue to influence perceptions of racial difference. Past stereotypical themes such as the “black brute” and “noble savage” are evident in contemporary media depictions of black males as dangerous (e.g., notions of black men as thugs and gangsters) and superhuman (e.g., notions of what Spike Lee has termed “the mystical, magical negro” and the black athlete) [10–13]. Waytz, Hoffman, and Twawalter’s recent study [14] shows that a significant number of white persons still perceive black persons on both implicit and explicit levels to possess superhuman capabilities as well as deny blacks persons’ pain. This research along with Wilson’s testimony highlights the importance of analyzing the intersection of suspects’ race and size when studying police behavior.

Another recent high profile case suggesting a connection between race, perceived size, and police treatment involved the New York Police Department (NYPD) and 6’7, 220 lbs., black National Basketball Association (NBA) player, Thabo Sefolosha, who suffered a broken fibula in NYPD custody on April 9^th^, 2015 while his teammate and fellow-arrestee, 6’11, 260 lbs., Pero Antic, who is white, was unharmed [15]. The NYPD provides a rich ground for investigation into racial disparities in policing because of their Stop, Question, and Frisk program. The NYPD Stop, Question, and Frisk program is legally rooted in *Terry v*. *Ohio* (1968) [16], which permits law enforcement officers to conduct limited warrantless searches if they have a level of suspicion of probable cause. Under *Terry*, if officers witness unusual conduct which leads them to believe that a person has a weapon and is presently dangerous, officers may conduct a search and frisk the individual to determine if they are carrying a weapon. Although The Fourth Amendment [17] protects citizens against unreasonable searches and seizures and requires search warrants to be judicially sanctioned and supported by probable cause, in 2009, the Wisconsin Court of Appeals upheld that a suspect’s furtive-type movement (suspicious, shifty, or stealthy behavior), and other circumstances suggestive of a threat to a police officer’s safety, can be the basis for a protective search [18].

In Brownsville, Brooklyn, one of the precincts with the greatest percentage of stops by the NYPD as measured against its population (29.1% in 2011), furtive movement was listed by officers as the reason for half of the stops conducted between January 2006 and March 2010 [19,20]. Furtive movement is often discussed by scholars as a catch-all category for police to stop whomever they please, and frequently results in a disproportionate amount of racial minorities who are stopped. A 2012 report from The New York Civil Liberties Union [20] shows that in 70 of the 76 New York precincts, black and Hispanic persons were the suspects in over half of NYPD stops, even in areas where black and Hispanic individuals represented 14% or less of the population. In almost half (33) of the precincts, black and Hispanic persons accounted for more than 90% of stops and were more likely to be frisked than Whites who were stopped. The report also showed that in a one year period there were more stops of young black males relative to their population in the city, suggesting that young black men were stopped multiple times. Furthermore, 90% of the young black and Hispanic men who were stopped by NYPD officers were deemed to be innocent [21]. In Brownsville, the arrest rate between 2006 and 2010 was less than 1% compared to the 6% arrest rate in the rest of the city, and in more than 50,000 stops in Brownsville during this time period, only 25 guns were recovered [19,20]. Because of the empirical evidence documenting racial disparities in NYPD practices, Manhattan Federal Court Judge Shira Scheindlin stated in *Floyd v*. *City of NY* (2013) [22] that the NYPD had targeted racially defined groups, thus violating both the fourth and fourteenth constitutional amendments. However, Scheindlin’s ruling did not prohibit the use of Stop and Frisk tactics; rather, it required officers to more clearly prove the reason for their stops. It was not until March 2015 that NYPD officers were prohibited from conducting stops solely on the basis of suspects’ furtive movement or presence in a high crime area [23].

Although many studies [24–31] document the relationship between race and differential treatment by the NYPD, only one [29] examined the association between size, measured by body mass index (BMI; kg/m^2^), and criminal punishment by the NYPD. Masicampo et al. [29] found that rates of criminal punishment are related to suspects’ body size, suggesting the possibility that given the stigma of obesity, obese individuals are penalized more harshly for some types of crime. However, the authors did not report how race affected results, and by combining height and weight into a single index (BMI) lost the ability to estimate the separate associations of height and weight with criminal punishment. Cullinane et al. [32] examined the relationship between intimate partner violence, offender and victim body masses, and arrest decisions and found a significant correlation between offender and victim body masses and arrest decisions. However the researchers could not identify the cause of the correlation, and similarly to Masicampo et al., the study did not examine racial effects nor did it include a variable for height.

In the current study, we test the relationship between physical police intervention and both perceived size and BMI, as well as explore how suspects’ race may be associated with these relationships. Apart from Masicampo et al. and Cullinane et al., no previous research has analyzed suspects’ perceived body size and police practices, though past theories of biology and crime, such as Sheldon's somatotyping of criminal behavior [33] as well as several recent studies assess the relationship between individuals’ body type, height, and weight and likelihood of committing crime and being arrested [34–36]. Here we used data from the NYPD Stop, Question, and Frisk (SQF) Database, 2006–2013 to test whether perceived height and weight are related to physical police intervention, including whether suspects were frisked or searched, whether force was used against suspects, and whether race affects the relationship.

## Methods

### Data

NYPD SQF data are publically available and do not contain identifying information; therefore, this study was approved by The University of Alabama at Birmingham’s IRB under the Application for Designation of Not Human Subjects Research. The most recent years available for the NYPD SQF database are 2006–2013. Although data are available from 2003–2005, those years were excluded due to noted concerns with the usability of the coding of ‘suspected crime,’ a key covariate in our model.

The SQF database is comprised of officer-recorded reports of their interactions with individuals “when the officer has *reasonable suspicion* that a person is involved in criminal activity.” This level of interaction is referred to as a “stop, question, and frisk” interaction and is considered to be between “common-law right of inquiry” and arrest in terms of severity, although a SQF interaction may escalate into an arrest [30]. Each entry contains information regarding the suspect, the reasons for the stop, the circumstances of the stop, and the officer’s response. The database contains data from 4,111,828 stops; of those we analyzed the 3,195,304 stops of males 18 years and older, of which 10% were white, 53% were black, and 32% were Hispanic. Due to the small numbers of suspects denoted as Asian/Pacific Islander, American Indian/Alaskan native, and other (178,113 cases, 5% of sample) the three groups were merged and analyzed in a single ‘other’ category. Additional descriptive statistics for this sample are given in Table 1.

**Table 1:** Descriptive statistics for the values reported in the NYPD SQF database from 2006–2013. Categorical variables are given as N(%) while continuous variables are given as Median (IQR).

	Total	White	Black	Hispanic^1^
Sample Size	3,195,304	316,664 (10%)	1,681,834 (53%)	1,018,693 (32%)
Suspect Characteristics				
Age^2^	26 (21–37)	27 (21–39)	27 (21–37)	26 (21–35)
Height < 66”	316,284 (10%)	22,001 (7%)	106,530 (6%)	164,093 (16%)
66” ≤ Height < 73”	2,504,488 (78%)	254,507 (80%)	1,312,885 (78%)	795,771 (78%)
73” ≤ Height	374,532 (12%)	40,156 (13%)	262,419 (16%)	58,829 (6%)
Weight < 141 lbs.	319,388 (10%)	27,841 (9%)	141,321 (8%)	124,255 (12%)
141 lbs. ≤ Weight < 205 lbs.	2,552,680 (80%)	254,338 (80%)	1,350,522 (80%)	808,023 (79%)
205 lbs. ≤ Weight	323,236 (10%)	34,485 (11%)	189,991 (11%)	86,415 (8%)
Obese^2^ (BMI≥30)	308,946 (10%)	31,201 (10%)	154,256 (9%)	109,425 (11%)
Stop Time and Location				
High-Crime Time	1,244,406 (39%)	121,414 (38%)	674,908 (40%)	385,273 (38%)
High-Crime Location	1,828,742 (57%)	170,008 (54%)	991,425 (59%)	572,570 (56%)
Bronx	531,245 (17%)	17,301 (5%)	256,253 (15%)	237,476 (23%)
Brooklyn	1,086,850 (34%)	102,741 (32%)	705,463 (42%)	239,499 (24%)
Manhattan	710,959 (22%)	67357 (21%)	368,482 (22%)	241,624 (24%)
Queens	727,040 (23%)	79,506 (25%)	297,018 (18%)	270,486 (27%)
Staten Island	139,118 (4%)	49,748 (16%)	54,574 (3%)	29,573 (3%)
Police Intervention				
Frisked/Searched	1,770,432 (55%)	139,378 (44%)	958,218 (57%)	588,551 (58%)
Force Used	710,013 (22%)	53,815 (17%)	376,677 (22%)	246,390 (24%)
Weapon Drawn/Pointed	13,796 (0.43%)	1,288 (0.41%)	7,931 (0.47%)	3,849 (0.38%)

^1^ Includes Black-Hispanic and White-Hispanic individuals.

^2^ Includes only subjects with plausible values (N = 3,179,760 for age, N = 3,174,971 for obesity status). See supplementary material for details.

### Variable Definitions

We emphasize that when we refer to height, weight, or size, we refer to the officer-perceived values unless otherwise noted. We considered these perceived values to be reasonable for analysis as it is the officer’s perception of suspect size rather than clinically measured values that could affect their decision to physically intervene. The perceived size of the suspect was defined categorically based on the combination of recorded height (under 66”, 66–72”, ≥73”; under 5’6”, 5’6”-6’, ≥6’1”) and weight (under 141 lbs., 141–205 lbs., ≥205 lbs.). These bounds were chosen because they were the 10^th^ and 90^th^ percentiles in this sample and approximate reasonable bounds for what constitutes a tall, short, light, or heavy adult American male.

We also analyzed the NYPD SQF database using the suspects’ BMI category instead of height and weight categories. Suspect BMIs were calculated via the officer reported height and weights and categorized as underweight (under 18.5 kg/m^2^), normal weight (18.5 to 25 kg/m^2^), overweight (25 to 30 kg/m^2^), or obese (over 30 kg/m^2^). No values were missing but a small portion (0.64%) had values for height, weight, or BMI that were deemed biologically implausible based on what was observed in the National Health and Nutrition Examination Survey (NHANES) from 2005–2012 [37]. Suspects with implausible anthropometric values had their BMI replaced using multiple imputation [38].

### Statistical Analysis

Generalized linear mixed models were used to make inferences about the combinations of race and size, modeled as a race-by-height-by-weight interaction, with regard to the marginal odds of the suspect being frisked or searched and to the odds of having force used against them. The estimated adjusted odds ratios were calculated with medium-sized (66–72”, 141–205 lbs.) white suspects as the reference group while controlling for thirty-two covariates relating to the time and place of the stop, the reasons for the stop (including the suspected crime), and suspect characteristics besides race and size [30]. The precinct in which the stop took place was treated as a random effect to account for correlation present between stops due to similarities in the neighborhoods or the officers making the stops. The covariates used in the analysis are listed in Table A in S1 File, and their effects are given in Tables B, C, D, and E in S1 File. The alpha level to determine statistical significance and confidence interval (CI) width was set at 0.01 (2-tailed; 99% CIs) to account for the large sample size.

The analysis of the different definition of size using BMI categories was identical to the one described above. Full methodological details are available in the supplement (S1 File).

## Results

### Suspects’ Perceived Height, Weight, Race, and Police Physical Intervention

**Fig 1:**
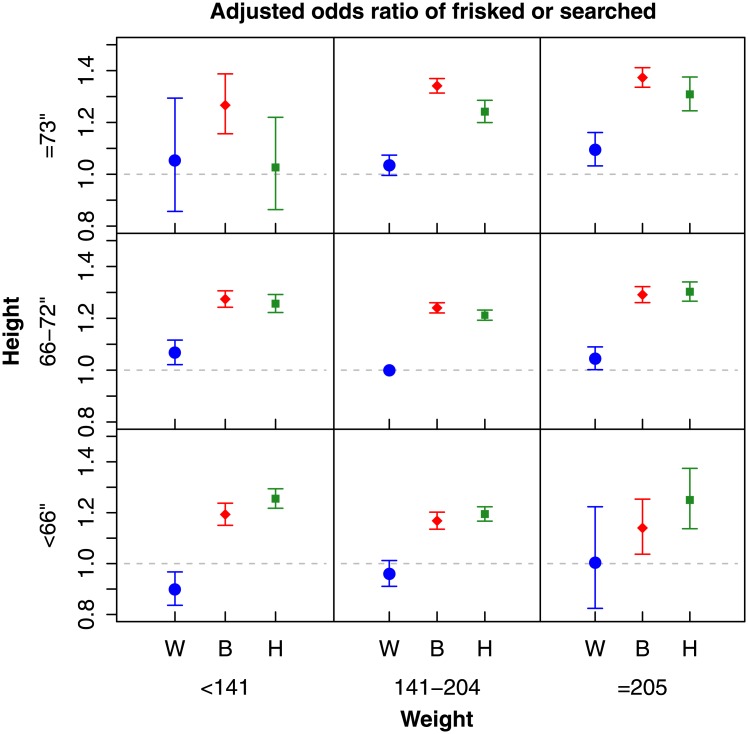
The adjusted odds ratios for the suspect being frisked or searched by race, height, and weight category compared to white suspects with weight 141–204 pounds and 66–72” in height, controlling for stop characteristics. The blue circle denotes white (W) suspects, the red diamond denotes black (B) suspects, and the green square denotes Hispanic (H) suspects. Error bars indicate the 99% confidence interval for the group estimates.

Results indicated that for most height and weight categories, black and Hispanic suspects were at increased risk of being frisked or searched compared to their white counterparts, even when controlling for the circumstances of the stop (Fig 1). The counts and proportions of stops with physical intervention for each height/weight/race category are given in Table 2.

**Table 2:** The number of stops for each of the height/weight/race categories, and the number (%) of stops involving frisks/searches and uses of force.

Race	Height	Weight	# Stops	# Frisks/Searches (%)	# Force Used (%)
White	<66”	<141	7,170	2,833 (39.5%)	1,119 (15.6%)
		141–205	13,899	5,808 (41.8%)	2,125 (15.3%)
		>205	932	369 (39.6%)	141 (15.1%)
	66–72”	<141	19,846	8,850 (44.6%)	3,342 (16.8%)
		141–205	212,035	93,280 (44.0%)	35,739 (16.9%)
		>205	22,626	9,869 (43.6%)	4,048 (17.9%)
	≥73”	<141	825	371 (45.0%)	134 (16.2%)
		141–205	28,404	12,931 (45.5%)	5,103 (18.0%)
		>205	10,927	5,067 (46.4%)	2,064 (18.9%)
Black	<66”	<141	35,967	19,370 (53.9%)	7,651 (21.3%)
		141–205	66,099	35,428 (53.6%)	13,953 (21.1%)
		>205	4,464	2,305 (51.6%)	935 (21.0%)
	66–72”	<141	100,453	57,254 (57.0%)	22,338 (22.2%)
		141–205	1,100,925	628,585 (57.1%)	243,844 (22.2%)
		>205	111,507	61,888 (55.5%)	25,522 (22.9%)
	≥73”	<141	4,901	2,748 (56.1%)	1,068 (21.8%)
		141–205	183,498	107,660 (58.7%)	43,508 (23.7%)
		>205	74,020	42,980 (58.1%)	17,858 (24.1%)
Hispanic	<66”	<141	52,737	29,905 (56.7%)	13,128 (24.9%)
		141–205	106,956	59,236 (55.4%)	25,617 (24.0%)
		>205	4,400	2,435 (55.3%)	1,043 (23.7%)
	66–72”	<141	70,204	40,843 (58.2%)	17,208 (24.5%)
		141–205	661,120	383,322 (58.0%)	158,406 (24.0%)
		>205	64,447	37,691 (58.5%)	16,052 (24.9%)
	≥73”	<141	1,314	709 (54.0%)	325 (24.7%)
		141–205	39,947	23,713 (59.4%)	9,972 (25.0%)
		>205	17,568	10,697 (60.9%)	4,639 (26.4%)

Among medium-sized (66–72”, 141–205 lbs.) suspects, black suspects had 24.0% increased odds of being frisked/searched (99% CI: 22.1% to 26.0%) and Hispanic suspects had 21.2% increased odds (99% CI: 19.3% to 23.2%) compared to medium-sized white suspects. The height-weight category was also observed to have a significant association with the odds of being frisked/searched (p<0.0001) such that large suspects had higher odds of being frisked/searched. White suspects perceived as very large (≥73”, over 205 lbs.) had 9.5% higher odds of being frisked/searched compared to medium-sized white suspects (99% CI: 3.2% to 16.1%). This increase was even more pronounced in very large black (37.3%, 99% CI: 33.6% to 41.2%) and Hispanic (30.9%, 99% CI: 24.5% to 37.6%) suspects when compared to medium-sized white suspects. Predicted probabilities of being frisked or searched are given in Figure A in S1 File.

**Fig 2:**
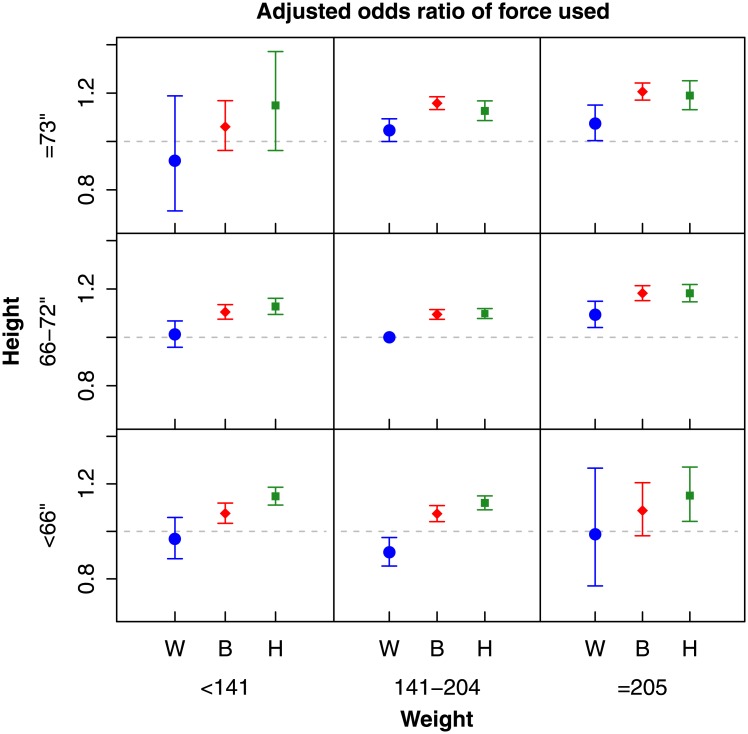
The adjusted odds ratios for the suspect having force used on them by race, height, and weight category compared to white suspects with weight 141–204 lbs. and 66–72” in height, controlling for stop characteristics. The blue circle denotes white (W) suspects, the red diamond denotes black (B) suspects, and the green square denotes Hispanic (H) suspects. Error bars indicate the 99% confidence interval for the group estimates.

The finding of non-white and large suspects experiencing more physical intervention by the NYPD was also seen when we examined whether the officer reported force being used on the suspect (Fig 2). Among medium-sized (66–72”, 141–205 lbs.) suspects, black suspects had 9.5% increased odds (99% CI: 7.5% to 11.5%) and Hispanic suspects had 9.8% increased odds (99% CI: 7.8% to 11.9%) of having force used on them compared to medium-sized white suspects.

White suspects perceived as very large (73” and over, over 205 lbs.) had 7.4% higher odds of having force used on them compared to medium-sized white suspects (99% CI: 0.3% to 15.0%). Very large black (20.6%, 99% CI: 17.1 to 24.2%) and Hispanic (19.0%, 99% CI: 13.2% to 25.1%) suspects had the greatest odds of having force used upon them compared to medium-sized whites. Predicted probabilities of having force used are given in Figure B in S1 File.

### Suspects’ BMI, Race, and Physical Police Response

The observed number of stops with physical intervention within each race-by-BMI category group is listed in Table 3, and the predicted probabilities are given in Figure C in S1 File.

**Table 3:** The number of stops for each of the BMI category-by-race groups, and the number (%) of stops involving frisks/searches and uses of force.

Race	BMI Category	# Stops	# Frisks/Searches (%)	# Force Used (%)
White	Underweight	3,253	1,376 (42.3%)	505 (15.5%)
	Normal weight	143,022	65,050 (45.5%)	25,100 (17.6%)
	Overweight	138,912	59,438 (42.8%)	22,831 (16.4%)
	Obese	31,477	13,514 (42.9%)	5,379 (17.1%)
Black	Underweight	16,125	8,945 (55.5%)	3,487 (21.6%)
	Normal weight	788,282	461,331 (58.5%)	181,984 (23.1%)
	Overweight	721,882	402,470 (55.8%)	156,179 (21.6%)
	Obese	155,545	85,472 (55.0%)	35,027 (22.5%)
Hispanic	Underweight	7,239	4,079 (56.4%)	1,744 (24.1%)
	Normal weight	426,890	251,224 (58.9%)	105,521 (24.7%)
	Overweight	474,394	270,809 (57.1%)	112,704 (23.8%)
	Obese	110,170	62,439 (56.7%)	26,421 (24.0%)

**Fig 3:**
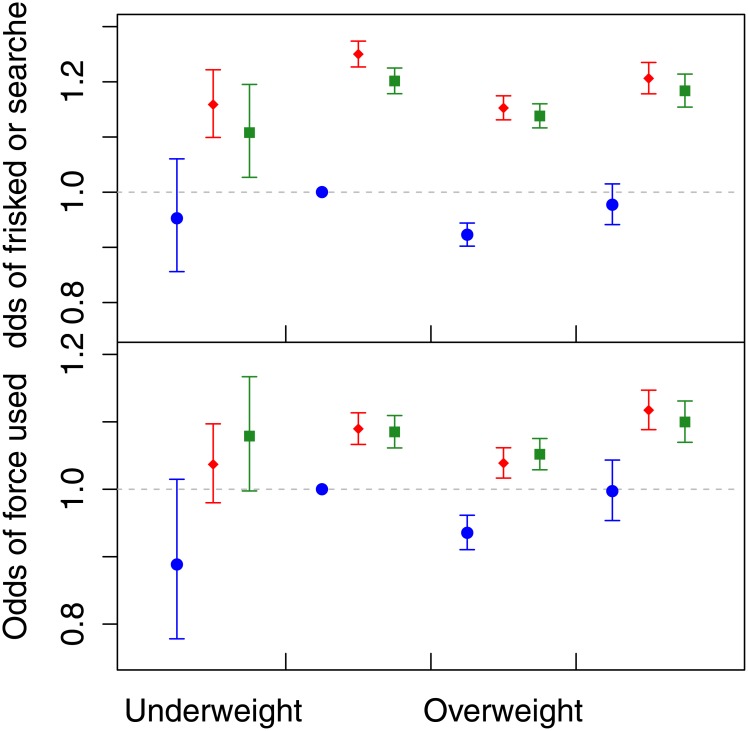
The adjusted odds ratios for the suspect being frisked or searched (top) or having force used on them (bottom) by race and BMI category compared to normal weight white suspects, controlling for stop characteristics. The blue circle denotes white suspects, the red diamond denotes black suspects, and the green square denotes Hispanic suspects. Error bars indicate the 99% confidence interval for the group estimates.

When modeling the odds of being frisked/searched versus race and BMI category (Fig 3, top), we estimated that normal weight black (24.9%, 99% CI: 22.6% to 27.3%) and Hispanic (20.1%, 99% CI: 17.8% to 22.4%) suspects had increased odds compared to normal weight white suspects.

Looking within race categories, we did not find evidence that obese white (-2.2%, 99% CI: -5.8% to +1.5%) or Hispanic (-1.4%, 99% CI: -3.5% to 0.6%) suspects had qualitatively different odds of being frisked/searched compared to their normal weight counterparts. We did observe a small decrease in the odds of being frisked or searched for obese black suspects (-3.6%, 99% CI: -5.3% to -1.9%) compared to normal weight black suspects.

When examining the odds of force being used (Fig 3, bottom) we see that obese black (11.7%, 99% CI: 8.8% to 14.7%) and Hispanic (10.0%, 99% CI: 7.0% to 13.1%) suspects have the greatest odds compared to normal weight whites, slightly higher than normal weight black (9.0%, 99% CI: 6.7% to 11.3%) and Hispanic (8.5%, 99% CI: 6.1% to 10.9%) suspects. When looking within-race categories the odds of force being used significantly increased from normal weight to obese for black suspects (2.5%, 99% CI: 0.7% to 4.4%) but not for white (-0.3%, 99% CI: -4.6% to 4.3%) or Hispanic suspects (1.4%, 99% CI: 1.4% (-0.8% to 3.6%).

## Discussion

Similarly to police forces across the country, the mission of the New York City Police Department (NYPD) is to “… enforce the laws, preserve the peace, reduce fear, and provide a safe environment” where police pledge to “… impartially enforce the law… value human life, respect the dignity of each individual and render (their) services with courtesy and civility.” [39] However, while police do much good, in some instances, the very people who are employed to serve and protect citizens sometimes perpetuate violence upon those same citizens. Recent events have drawn public awareness to issues of police violence, and specifically, the relationship between citizens’ race and officers’ behavior. Our results add to the discussion by showing that not only are there racial disparities in police physical intervention, there is also an association between suspects’ officer-perceived size and police physical intervention as well as an association between suspects’ race, their officer-perceived size, and police treatment. Although suspects perceived to be tall *or* heavy have increased risk of physical police punishment, suspects perceived as tall *and* heavy are at still greater risk and tall, heavy, black and Hispanic men are at the greatest risk of all.

Further analyses show that there is a slight association between suspects’ obesity status and officers’ decisions to physically intervene, especially for obese black suspects. In fact, Garner, Brown, and Parker, were all classifiable as obese (BMIs were 30 or more) [1,7,8] at the time of their deaths. However, the associations are not monolithic and do not lend themselves to obvious interpretation. More work is needed to explore whether obesity status as a function of race affects officers’ decisions. Obesity may be an important component in studying police treatment of suspects not only because obesity had risen nationwide, but because obesity is more common in certain racial groups [40], and is a highly stigmatized condition [41–43]. Past research shows that rates of weight discrimination are high [44] and that obesity elicits stigma and negative character assessments of obese people as non-compliant, evil, disgusting, unintelligent, and mean-spirited [44–51] which may affect police behavior.

Aside from police treatment, recent studies [52,53] on size and the criminal justice system have focused on sex but not race. The current study is the first to test the interaction among race, perceived size, and criminal punishment. Although it has been shown that even when men and women are of equal actual height, men are perceived to be taller than women [54], we know of no comparable findings on the effects of race on perceived body size and conducting such studies would seem merited.

Our results suggest that researchers should incorporate measures of suspects’ size (or perhaps more aptly, perceived size) when studying police behavior and police may benefit from information about stereotypes associated with size and race. To explore and address long-standing issues of racial stereotyping and bias, future research should examine how officers perceive the sizes of differently-raced suspects as well as their feelings towards differently-raced suspects across size categories. One weakness of our study is that the data are merely observational, which inhibits our ability to assert that the association between race/size and stop outcomes is causal. Experiments should be conducted to determine if there is an association between suspects’ race and police officers’ likelihood of overestimating or underestimating suspects’ size. Another weakness of our study is that it only examines male suspects. Future studies should examine the relationship between race, perceived size, and physical police intervention with regard to female suspects in order to understand the nuances associated with intersectionality in terms of race, perceived size, sex, and police treatment. Additionally because our study only utilized NYPD data, our findings cannot necessarily be generalized to the broader population, and research examining suspects’ race, perceived size, and differential treatment by police would benefit from nationally representative data.

To reduce the influence of both covert and overt prejudice in police treatment of suspects, we recommend that officer training programs incorporate education on different types of racial bias which extend beyond old-fashioned, biological, Jim Crow racism to include instruction on cultural and more nuanced forms of racism such as systemic racism [55], symbolic racism [56], laisse-faire racism [57], and colorblind racism [58]. We also encourage policy makers and scholars to continue to examine how legislation associated with racial profiling may result in disparities and how the personal characteristics of police officers and suspects are related to inequalities in the criminal justice system.

## Supporting Information

S1 File This file contains additional details of the materials and statistical methods used and associated references. It contains tables containing the covariates used (Table A) and their effects on the odds of frisk/search and force used (Tables B, C, D, and E). It also contains the specific parameter estimates for the race-by-size predictors in the model (Tables F, G, H, and I) and the predicted probabilities of those groups (Figures A, B, and C).
(PDF)
