# Supplementary material for: Black and Hispanic Men Perceived to Be Large Are at Increased Risk for Police Frisk, Search, and Force
Source: PLoS One. 2016 Jan 19;11(1):e0147158. doi: 10.1371/journal.pone.0147158 (PMC4718646; doi:10.1371/journal.pone.0147158)
Supplement: S1 File — It contains tables containing the covariates used (Table A) and their effects on the odds of frisk/search and force used (Tables B, C, D, and E). It also contains the specific parameter estimates for the race-by-size predictors in the model (Tables F, G, H, and I) and the predicted probabilities of those groups (Figures A, B, and C). (PDF) [file pone.0147158.s001.pdf]

Black and Hispanic Men Perceived to be Large Are At-Risk for Police Frisk, Search, and Force

Authors: Adrienne N. Milner, Brandon J. George, David B. Allison

Supporting Information:

Materials and Methods: The detailed description of the data and analyses used in this study.

Table A: A list of the stop characteristics used as covariates in this study along with their use in past studies on the NYPD SQF data.

Table B: Effects of the suspected crime on the odds of the suspect being frisked/searched or having force used on him.

Table C. Effects of the time of the stop on the odds of the suspect being frisked/searched or having force used on him.

Table D. Effects of the location of the stop on the odds of the suspect being frisked/searched or having force used on him.

Table E. Effects of other circumstances of the stop on the odds of the suspect being frisked/searched or having force used on him.

Table F. Effects of race, height, and weight on the odds of the suspect being frisked/searched.

Table G. Effects of race, height, and weight on the odds of the suspect having force used on him.

Table H. Effects of race and BMI category on the odds of the suspect being frisked/searched.

Table I. Effects of race and BMI category on the odds of the suspect having force used on him.

Figure A. Predicted probabilities of being frisked or searched for the "average" suspect of each height, weight, and race group.

Figure B. Predicted probabilities of having force used for the "average" suspect of each height, weight, and race group.

Figure C. Predicted probabilities of physical intervention for the "average" suspect of each race and BMI category.

Materials and Methods

The database used data from the New York Police Department (NYPD) Stop, Question, and Frisk (SQF) Database, 2006-2013 to explore whether perceived height and weight are related to physical police intervention, including whether suspects were frisked or searched, whether force was used against suspects, and how race is associated with this relationship. The SQF database is comprised of officer-recorded reports of their interactions with individuals "when the officer has *reasonable suspicion* that a person is involved in criminal activity." This level of interaction is referred to as a "stop, question, and frisk" interaction and is considered to be between

"common-law right of inquiry" and arrest in terms of severity, although a SQF interaction may escalate into an arrest. The 2008 report by Ridgeway on behalf of the RAND Corporation goes into great detail as to how these SQF interactions are recorded and validated, so we will refer interested readers to his descriptions [30]. Note that although NYPD SQF data is available from 2003 to 2005, those years were excluded due to noted concerns with the usability of 'suspected crime,' a key covariate in our model.

The data used in this paper are publically available from the NYPD Stop, Question, and Frisk Report Database available: www.nyc.gov/html/nypd/html/analysis_and_planning/stop_question _and_frisk_report.shtml. Additional information regarding the content of the database has been archived by the University of Michigan's Inter-university Consortium for Political and Social Research (ICPSR) available: http://www.icpsr.umich.edu/icpsrweb/NACJD/studies/21660.

The core of our analysis were generalized linear mixed models that estimated the odds of two outcomes, analyzed in separate models; the first was whether suspects were frisked or searched, and the second was whether suspects had some measure of force used against them. Types of force used included putting the suspect on the ground, putting the suspect against a wall, drawing a weapon, pointing a weapon at the suspect, using a baton, using handcuffs, using pepper spray, 'hands,' or 'other.' In this analysis we focused on the 3,195,304 males recorded as 18 years or older out of the 4,111,828 total stops recorded. The foci of inference were the crossed categories of race and size or race and body mass index (BMI) category, defined below. Due to the large sample size, we considered 99% confidence intervals around the estimated odds ratios of each race-by-size category. Inspired by Ridgeway's method of analyzing this data, we used a wide variety of covariates so that differences in the odds due to race or size/obesity would be less likely to be due to possible confounders. The covariates used are given in Table S1, along with their historical use in other studies that have analyzed this dataset. Furthermore, the precinct of the stop was treated as a random effect to control for observed correlation between stops, due to spatial proximity or from having similar groups of patrolling officers. The analysis was done using PROC GLIMMIX in SAS 9.4 (SAS Institute Inc., Cary, NC), and predicted probabilities of physical

intervention were calculated using its LSMEANS statement with 'average' covariate levels specific to each race/size group.

As the NYPD SQF database is extremely large, it is not surprising that there existed some missing data or potentially erroneous values. To the NYPD's credit, the actual amount of missing or problematic values was impressively small (<2%) and generally pertained to more subjective fields such as suspect age, height or weight. There were no missing values for whether the suspect was frisked, searched, or had some type of force used on them. In general, missing or questionable values were imputed using fully conditional specification in PROC MI in SAS 9.4, but details for specific predictors and covariates are given below. We considered it important to include all recorded stops to reduce any possible selection bias due to missing values.

It should be noted that the SQF database contained no information about the reporting officer beyond an identification number of the officer's command. Therefore, we were unable to control for or make inferences about the effects of the officer's race, size, or gender. We acknowledge that those are likely predictors of physical intervention and may interact with the suspect's race and size, but a lack of data precludes their inclusion in our analysis.

For race, there were no values explicitly missing although 50,459 (1.58% of sample) were listed as 'other.' The other codings in the database included White, Black, White-Hispanic, Black-Hispanic, Asian/Pacific Islander, and American Indian/Alaskan native. As there was no qualitative difference in outcomes or covariates between White- and Black-Hispanics, the two were merged and analyzed as a single 'Hispanic' category. Due to the small numbers of Asian/Pacific Islander, American Indian/Alaskan native, and other (178,113 cases, 5.57% of sample) the three were merged and analyzed in a single 'other' category.

For anthropometric variables such as height and weight (and the resulting BMI) there were no missing values. However, there were numerous (20,333 cases, 0.64% of sample) values that seemed unreasonable (e.g. weights of 0 or 17,000 pounds, point masses for height at 36" and 95"). In order to make an objective distinction between values that were implausible and merely unlikely, we considered the National Health and Nutrition Examination Survey (NHANES) data from 2005-2012 [37]. We pooled the data for males 18 and

older from those eight years and observed the ranges of measured heights to be (54.9”, 80.5”), weights to be (87.3 lbs., 817.9 lbs.), and BMIs to be (14.2 kg/m^2, 130.21 kg/m^2). For the analysis of obesity categories, subjects who had any anthropometric values outside the range seen in NHANES were considered implausible, and their values for BMI were imputed. After all suspects had plausible BMI values, they were categorized as underweight (under 18.5 kg/m^2), normal weight (18.5 to 25 kg/m^2), overweight (25 to 30 kg/m^2), or obese (over 30 kg/m^2). For the size categorization, we considered the 10th and 90th percentiles for height (66”,73”) and weight (141 lbs., 205 lbs.), regardless of the plausibility of the values. We considered these bounds acceptable as they are reasonable cutoffs for what one may consider an adult American male as tall or short, and light or heavy. Our rationale for using the raw officer-reported values in this analysis is because we hypothesize that it is the officer’s perception of suspect size that drives their actions towards the suspect; if the officer is going to record an implausible value for height or weight, we believe it is more likely that they will record an implausibly high value for a large suspect and an implausibly low value for a suspect perceived as small. Furthermore, a sensitivity analysis was performed where suspects with implausible values were omitted, and there was no meaningful effect on the estimates. There are some additional concerns with the recording of height and weight that we could not account for in our analysis, however. It is unknown how much the recorded values are truly the officer’s perception and how much they may be self-reported by the suspect (which introduces possible biases) or from the suspect’s identification (which may also contain biases). The height and weight values may be subject to recall bias when filled out by the officer after the stop; this is potentially problematic if recall is affected by the outcome of the stop (e.g. the officer records a suspect as being larger if force was used).

For age, we only considered stops where the suspect was listed as 18 years or older. However, there were 15,544 (0.49% of sample) values where the suspect was listed as being 99 or older, with a point mass at 99. These values were considered to be implausible, and values given as 99 or older were deleted and replaced with imputed values. Also, in the analysis the square of age was included with the linear term as a covariate, given the well-established non-monotonic relationship of age with crime.

For crime suspected, all crime codes with at least 1000 recorded cases were included in the analysis as a specific category. This included 36 specific crimes, from 836,423 cases of criminal possession of a weapon to 1129 cases of forcible touching. The 25,280 (0.79% of sample) stops where the suspected crime was either missing or had under 1000 reported cases were combined into an ‘other’ category. This was considered reasonable as the ‘other’ category had outcome rates at approximately that of the total sample. Furthermore, we potentially reduce the bias in estimating the associations of suspected crime with the outcomes that may arise from combining crimes based on a subjective criteria (i.e. all drug crimes, all violent crimes).

For crime location, there were a small number of cases where the precinct or X/Y coordinates were missing, but not both. When the precinct was missing, it was manually imputed based on the precinct observed with other stops in the same X/Y coordinate region. When the X/Y coordinates were missing, the average coordinates of that stop’s precinct were used.

For the indoor/outdoor location, type of suspect identification provided, month, day of the week, time of day, and whether the officer was in uniform all had a small number of missing values that were handled with multiple imputation.

Lastly, we omitted covariates that were identified as a reason for frisking or a basis for searching. This included suspect non-compliance with officer directions and verbal threats made by the suspect, both of which were used as covariates in previous studies [59-61]. These variables were excluded as they were only indicated in the presence of a frisk or search, and approximately never when neither was performed. Therefore these covariates were redundant when frisk/search was the outcome and caused confusion with the causal pathway when force was the outcome.

Additional Results

Perceived Height, Weight, and the Odds of Frisking or Searching

The results for the parameters for race and height/weight category when modeling for the odds of frisking or searching are given in Table S6. The race-by-height-by-weight interaction term was significant

($F_{24,3.2*10^6} = 5.32, p < 0.0001$), with significant positive interactions for blacks over 74” and between 141-206 lbs. ($t_{3.2*10^6} = 2.80, p = 0.0051$) and Hispanics under 66” and under 141 lbs. ($t_{3.2*10^6} = 4.65, p < 0.0001$). The overall main effects for race ($F_{3,3.2*10^6} = 96.18, p < 0.0001$) and height-weight category ($F_{8,3.2*10^6} = 33.15, p < 0.0001$) were strongly significant, as were the parameters for black ($t_{3.2*10^6} = 34.78, p < 0.0001$), Hispanic ($t_{3.2*10^6} = 30.63, p < 0.0001$), and tall-and-heavy (over 74”, over 205 lbs.) suspects ($t_{3.2*10^6} = 3.97, p < 0.0001$). This suggests that although race and perceived size do not have a clear pattern of interaction in their effects on the odds of being frisked or searched, the two factors are still additive such that large black and Hispanic suspects are at the greatest risk.

Perceived Height, Weight, and the Odds of Force Being Used

The results for the parameters for race and height/weight category when modeling for the odds of force being used are given in Table S7. The race-by-height-by-weight interaction term was significant ($F_{24,3.2*10^6} = 3.01, p < 0.0001$), although the only significant parameter was for Hispanics under 66” and between 141-205 lbs. ($t_{3.2*10^6} = 4.15, p < 0.0001$). The overall main effects for race ($F_{3,3.2*10^6} = 29.93, p < 0.0001$) and height-weight category ($F_{8,3.2*10^6} = 20.54, p < 0.0001$) were strongly significant, as were the parameters for black ($t_{3.2*10^6} = 12.62, p < 0.0001$), Hispanic ($t_{3.2*10^6} = 12.90, p < 0.0001$), and tall-and-heavy (over 74”, over 205 lbs.) suspects ($t_{3.2*10^6} = 2.70, p = 0.0069$). As with frisking, this suggests that the effects of race and size on the use of force are additive but not necessarily synergistic.

Perceived BMI and the Odds of Frisking or Searching

The results for the parameters for race and BMI category when modeling for the odds of frisking or searching are given in Table S8. We found that despite the race-by-BMI category interaction being significant ($F_{9,3.2*10^6} = 4.21, p < 0.0001$) none of the terms involving black or Hispanic suspects were individually significant at a 0.01 level. We again observed that blacks ($t_{3.2*10^6} = 30.73, p < 0.0001$) and Hispanic ($t_{3.2*10^6} = 24.39, p < 0.0001$) suspects are at greater risk compared to Whites and that this trend holds across all BMI categories (Fig. 3). Overweight white suspects had significantly lower ($t_{3.2*10^6} = 9.13, p < 0.0001$)

odds of being frisked or searched than normal weight white suspects, but this did not hold for obese white suspects ($t_{3.2*10^6} = 1.54, p = 0.1235$).

Perceived BMI and the Odds of Force Being Used

The results for the parameters for race and BMI category when modeling for the odds of force being used are given in Table S9. We observed that there was a significant race-by-BMI category interaction ($F_{9,3.2*10^6} = 2.43, p = 0.0094$) on the odds of force being used such that overweight Hispanic suspects were significantly more likely to have force used than race and BMI category alone would indicate ($t_{3.2*10^6} = 3.01, p = 0.0026$). The main effects for race ($F_{3,3.2*10^6} = 39.56, p < 0.0001$) and BMI category ($F_{3,3.2*10^6} = 37.02, p < 0.0001$) were strongly significant with black ($t_{3.2*10^6} = 10.29, p < 0.0001$) and Hispanic ($t_{3.2*10^6} = 9.49, p < 0.0001$) suspects at increased odds while white overweight ($t_{3.2*10^6} = 6.30, p < 0.0001$) suspects had decreased odds of having force used on them. Obese white suspects were not found to be at increased odds of force being used ($t_{3.2*10^6} = 0.15, p = 0.8825$).

References

59. Khruakham S, Hoover LT. The impact of situational and contextual factors on police arrest decisions: an analysis from the New York Police Department. Law Enforcement Executive Forum. 2012;12: 122-135.

60. Ferrandino J. The efficiency of frisks in the NYPD, 2004-2010. Criminal Justice Review. 2013;38: 149-168.

61. Lee J. Police use of nonlethal force in New York City: situational and community factors. Polic Soc. 2014; 1-14.

Class	Variable	Previous Covariate Use in Literature			
		Ridgeway 2008 [30]	Khruakham 2012 [59]	Ferrandino 2013 [60]	Lee 2014 [61]
Location of Stop	Precinct	X		X	
	Housing, Transit, or Neither	X	X		X
	Indoors/Outdoors.	X			
	X and Y coordinates of stop	X			
	Area with high incidence of crime	X	X		X
Time of Stop	Year	2006 only	2006 only	X	2006 only
	Month	X			
	Day of week	X			
	Time of day (4 hour blocks)	X	X		X
	Time of day with high-crime incidence	X			
Suspect characteristics and behavior	Age	X	X		X
	Type of identification provided	X			
	Carrying a suspicious object	X			
	Perceived to be casing a victim/location	X			
	Perceived to be acting as a lookout	X			
	Wearing clothes commonly used in a crime	X			
	Suspicious bulge	X			
	Evasive responses to questioning	X			
	Associating with known criminals	X			
	Furtive movements	X			
	Changed direction at sight of officer	X			
	Perceived actions indicative of a drug transaction	X			
	Perceived actions indicative of a violent crime	X			
Additional reasons for stop	Type of crime suspected	X			X
	Radio run	X			
	Report by victim, witness or officer	X	X		
	Ongoing investigation	X			
	Proximity to scene of a crime	X			
	Suspect fit a relevant description	X			
	Sights or sounds of criminal activity	X			
Officer behavior	Officer in uniform	X			

Table A. Covariates used in previous studies of the NYPD SQF dataset to control for stop characteristics.

Crime suspected	Number of stops	Number of frisk/searches (%)	Number of uses of force (%)	OR for frisk/search	OR for force used
Assault	101,825	59,380 (58.3%)	32,527 (31.9%)	1.49 (1.43,1.55)	1.25 (1.19,1.30)
Auto stripping	7,379	2,201 (29.8%)	1,115 (15.1%)	0.55 (0.51,0.60)	0.60 (0.55,0.66)
Burglary	345,280	144,120 (41.7%)	51,036 (14.8%)	1.02 (0.98,1.05)	0.72 (0.69,0.75)
Computer trespass	2,771	628 (22.7%)	238 (8.6%)	0.55 (0.48,0.62)	0.45 (0.37,0.54)
Crim. contempt	1,631	1,055 (64.7%)	570 (35.0%)	2.60 (2.25,3.01)	1.92 (1.66,2.22)
Crim. mischief	34,864	12,947 (37.1%)	6,142 (17.6%)	0.71 (0.68,0.75)	0.71 (0.68,0.75)
CP[1] controlled substance	109,186	46,321 (42.4%)	17,701 (16.2%)	0.93 (0.89,0.96)	0.72 (0.68,0.75)
CP forged instrument	5,366	1,930 (36.0%)	1,230 (22.9%)	0.63 (0.58,0.69)	0.86 (0.79,0.95)
CP marijuana	112,774	47,415 (42.0%)	18,924 (16.8%)	0.99 (0.95,1.03)	0.87 (0.83,0.91)
CP stolen property	22,327	6,823 (30.6%)	3,107 (13.9%)	0.62 (0.58,0.65)	0.62 (0.58,0.75)
CP weapon	836,423	740,344 (88.5%)	296,298 (35.4%)	6.61 (6.37,6.86)	1.61 (1.54,1.67)
CS[2] controlled substance	92,724	34,335 (37.0%)	14,464 (15.6%)	0.69 (0.67,0.72)	0.64 (0.61,0.67)
CS marijuana	9,805	4,649 (47.4%)	2,179 (22.2%)	1.01 (0.95,1.08)	0.99 (0.92,1.07)
Crim. tampering	4,216	1,044 (24.8%)	553 (13.1%)	0.41 (0.37,0.46)	0.49 (0.43,0.55)
Forcible touching	1,129	456 (40.4%)	304 (26.9%)	0.78 (0.66,0.92)	1.16 (0.97,1.40)
Forgery	6,556	2,516 (38.4%)	1,506 (23.0%)	0.63 (0.58,0.68)	0.80 (0.73,0.87)
Fraudulent accosting	4,482	936 (20.9%)	640 (14.3%)	0.47 (0.42,0.53)	0.60 (0.53,0.68)
Gambling	2,118	957 (45.2%)	474 (22.4%)	1.20 (1.06,1.36)	1.03 (0.89,1.20)
Graffiti	28,645	9,911 (34.6%)	3,897 (13.6%)	0.61 (0.58,0.64)	0.63 (0.60,0.67)
Grand larceny	139,544	59,915 (42.9%)	25,758 (18.5%)	0.78 (0.75,0.81)	0.72 (0.69,0.76)
Grand larceny auto	350,467	125,446 (35.8%)	51,867 (14.8%)	0.67 (0.64,0.69)	0.68 (0.65,0.71)
Harassment	1,560	516 (33.1%)	302 (19.4%)	0.71 (0.61,0.82)	0.75 (0.63,0.90)
Lewdness	2,937	735 (25.0%)	450 (15.3%)	0.38 (0.34,0.43)	0.58 (0.50,0.67)
Loitering	1,599	275 (17.2%)	142 (8.9%)	0.39 (0.33,0.47)	0.38 (0.30,0.49)
Menacing	3,253	2,029 (62.4%)	1,118 (34.4%)	2.39 (2.15,2.65)	1.51 (1.36,1.68)
Murder	1,540	952 (61.8%)	487 (31.6%)	2.08 (1.79,2.41)	1.42 (1.21,1.65)
Other	25,280	10,911 (43.2%)	5,878 (23.3%)	1	1
Petit larceny	77,159	21,605 (28.0%)	11,913 (15.4%)	0.45 (0.43,0.47)	0.59 (0.56,0.62)
Prostitution	4,552	955 (21.0%)	507 (11.1%)	0.34 (0.30,0.37)	0.42 (0.37,0.48)
Rape	4,005	1,830 (45.7%)	840 (21.0%)	0.89 (0.81,0.98)	0.73 (0.66,0.82)
Reckless endangerment	2,678	1,083 (40.4%)	630 (23.5%)	0.90 (0.80,1.01)	1.07 (0.94,1.22)
Robbery	520,599	344,108 (66.1%)	123,415 (23.7%)	2.09 (2.02,2.17)	1.05 (1.01,1.10)
Sexual abuse	4,988	1,506 (30.2%)	1,010 (20.3%)	0.43 (0.390.47)	0.70 (0.63,0.78)
Terrorism	4,106	546 (13.3%)	228 (5.6%)	0.34 (0.30,0.38)	0.28 (0.24,0.34)
Theft of services	9,250	2,884 (31.2%)	1,765 (19.1%)	0.61 (0.57,0.65)	0.79 (0.73,0.86)
Trademark counterfeiting	3,603	1,294 (35.9%)	879 (24.4%)	1.22 (1.10,1.35)	1.26 (1.13,1.41)
Trespass	308,683	75,874 (24.6%)	29,919 (9.7%)	0.63 (0.61,0.66)	0.56 (0.54,0.59)

[1]CP=Criminal Possession

[2]CS=Criminal Sale

Table B – Descriptive and inferential statistics relating the crime suspected by the officer prior to the stop to whether the suspect was frisked/searched or had force used on them. The adjusted odds ratios (OR) are for the suspected crime as a covariate in the model with the other covariates in Table S1 and the suspect’s anthropometric values categorized by height and weight (Size). The values are given as N(% of sample) or OR(99% confidence interval), and the adjusted odds are given with a suspected crime of “Other” as the reference group.

Covariate	Category	Number of stops	Number of frisk/searches (%)	Number of uses of force (%)	OR for frisk/search	OR for force used
Year	2006*	384,266	169,859 (44.2%)	78,881 (20.5%)	1	1
	2007	364,505	194,884 (53.5%)	85,229 (23.4%)	1.54 (1.52,1.57)	1.16 (1.14,1.18)
	2008	419,733	234,794 (55.9%)	101,210 (24.1%)	1.60 (1.58,1.63)	1.14 (1.12,1.16)
	2009	458,316	267,086 (58.3%)	115,052 (25.1%)	1.70 (1.67,1.72)	1.19 (1.17,1.21)
	2010	471,830	272,280 (57.7%)	111,482 (23.6%)	1.65 (1.62,1.67)	1.06 (1.04,1.07)
	2011	530,614	301,926 (56.9%)	116,854 (22.0%)	1.56 (1.54,1.58)	0.96 (0.94,0.97)
	2012	413,289	237,963 (57.6%)	73,102 (17.7%)	1.64 (1.62,1.66)	0.70 (0.69,0.72)
	2013	152,751	91,640 (60.0%)	28,203 (18.5%)	1.86 (1.83,1.90)	0.73 (0.71,0.74)
Month	January*	328,513	179,506 (54.6%)	71,651 (21.8%)	1	1
	February	305,294	164,849 (54.0%)	65,578 (21.5%)	1.00 (0.99,1.02)	1.00 (0.98,1.02)
	March	314,905	170,034 (54.0%)	68,398 (21.7%)	0.98 (0.96,0.99)	0.98 (0.97,1.00)
	April	295,670	162,127 (54.8%)	66,326 (22.4%)	0.96 (0.95,0.98)	0.99 (0.98,1.01)
	May	288,409	156,961 (54.4%)	64,558 (22.4%)	0.91 (0.89,0.92)	0.96 (0.95,0.98)
	June	240,066	131,523 (54.8%)	54,241 (22.6%)	0.89 (0.87,0.90)	0.95 (0.93,0.97)
	July	238,898	127,938 (54.5%)	52,989 (22.6%)	0.86 (0.84,0.88)	0.94 (0.92,0.95)
	August	242,265	134,035 (55.3%)	54,274 (22.4%)	0.89 (0.88,0.91)	0.91 (0.89,0.93)
	September	244,421	139,260 (57.0%)	54,719 (22.4%)	0.96 (0.94,0.98)	0.90 (0.88,0.92)
	October	265,594	153,268 (57.7%)	59,246 (22.3%)	1.03 (1.01,1.05)	0.91 (0.89,0.92)
	November	233,819	135,011 (57.7%)	53,390 (22.8%)	1.08 (1.06,1.10)	0.94 (0.93,0.96)
	December	201,450	115,920 (57.5%)	44,643 (22.2%)	1.10 (1.08,1.12)	0.93 (0.92,0.95)
Day of Week	Monday*	320,168	162,635 (50.8%)	65,530 (20.5%)	1	1
	Tuesday	464,152	261,371 (56.3%)	104,897 (22.6%)	1.09 (1.07,1.11)	1.05 (1.03,1.07)
	Wednesday	506,669	288,332 (56.9%)	114,072 (22.5%)	1.11 (1.09,1.13)	1.04 (1.03,1.06)
	Thursday	507,512	284,018 (56.0%)	113,577 (22.4%)	1.07 (1.06,1.09)	1.04 (1.03,1.06)
	Friday	530,355	299,255 (56.4%)	119,197 (22.5%)	1.07 (1.06,1.09)	1.03 (1.02,1.05)
	Saturday	503,114	283,046 (56.3%)	113,970 (22.7%)	1.03 (1.01,1.04)	1.02 (1.00,1.03)
	Sunday	363,434	191,775 (52.8%)	78,770 (21.7%)	0.98 (0.96,0.99)	1.00 (0.98,1.02)
Time of Day	12 AM – 4 AM	639,305	374,496 (58.6%)	155,711 (24.4%)	1.51 (1.49,1.53)	1.35 (1.33,1.37)
	4 AM – 8 AM	113,792	60,593 (53.3%)	28,870 (25.4%)	1.47 (1.44,1.50)	1.43 (1.40,1.47)
	8 AM – 12 PM*	279,322	115,887 (41.5%)	45,928 (16.4%)	1	1
	12 PM – 4 PM	532,113	276,744 (52.0%)	108,510 (20.4%)	1.18 (1.16,1.20)	1.14 (1.12,1.15)
	4 PM – 8 PM	683,836	373,691 (54.7%)	149,663 (21.9%)	1.28 (1.26,1.30)	1.20 (1.18,1.22)
	8 PM – 12 AM	946,936	569,021 (60.1%)	221,331 (23.4%)	1.42 (1.40,1.44)	1.25 (1.23,1.27)
High-crime time of day	Yes	1,244,406	703,121 (56.5%)	261,514 (21.0%)	1.01 (1.00,1.02)	0.91 (0.90,0.92)
	No*	1,950,898	1,067,311 (54.7%)	448,499 (23.0%)	1	1

Table C - Descriptive and inferential statistics relating covariates used in the model for the time of the stop to whether the suspect frisked/searched or had force used on them. The adjusted odds ratios (OR) are for the specific category of the covariate in the model with all the other covariates in Table S1 and the suspect's anthropometric values categorized by height and weight (Size). The values are given as N(% of sample) or OR(99% confidence interval), with the reference category denoted by *.

Covariate	Category	Number of stops	Number of frisk/searches (%)	Number of uses of force (%)	OR for frisk/search	OR for force used
Borough	Bronx	531,245	353,768 (66.6%)	164,082 (30.9%)	-	-
	Brooklyn	1,086,850	566,909 (52.2%)	189,033 (17.4%)	-	-
	Manhattan	710,959	355,895 (50.1%)	158,091 (22.2%)	-	-
	Queens	727,040	430,048 (59.2%)	171,360 (23.6%)	-	-
	Staten Island	139,118	63,756 (45.8%)	27,431 (19.7%)	-	-
Inside/ outside	Indoors	709,581	309,780 (43.7%)	131,379 (18.5%)	0.98 (0.96,0.99)	1.06 (1.05,1.08)
	Outdoors*	2,485,723	1,460,652 (58.8%)	578,634 (23.3%)	1	1
Housing/ transit	Housing	467,475	217,673 (46.6%)	64,267 (13.8%)	0.77 (0.76,0.78)	0.62 (0.61,0.63)
	Transit	249,522	129,361 (51.8%)	58,912 (23.6%)	1.52 (1.49,1.55)	1.27 (1.25,1.30)
	Neither*	2,478,307	1,423,398 (57.4%)	586,834 (23.7%)	1	1
High-crime area	Yes	1,828,742	995,683 (54.5%)	382,500 (20.9%)	0.63 (0.61,0.66)	0.63 (0.61,0.66)
	No*	1,366,562	774,749 (56.7%)	327,513 (24.0%)	1	1

Table D - Descriptive and inferential statistics relating covariates used in the model for the location of the stop to whether the suspect frisked/searched or had force used on them. The adjusted odds ratios (OR) are for the specific category of the covariate in the model with all the other covariates in Table S1 and the suspect’s anthropometric values categorized by height and weight (Size). The values are given as N(% of sample) or OR(99% confidence interval), with the reference category denoted by *.

Covariate	Category	Number of stops	Number of frisk/searches (%)	Number of uses of force (%)	OR for frisk/search	OR for force used
Type of ID provided	Photo*	1,846,741	1,004,036 (54.4%)	409,174 (22.2%)	1	1
	Verbal	1,243,247	713,398 (57.4%)	279,750 (22.5%)	0.94 (0.93,0.94)	0.93 (0.92,0.94)
	Other	44,217	23,085 (52.2%)	10,122 (22.9%)	1.05 (1.02,1.08)	1.06 (1.02,1.09)
	Refused	61,099	29,913 (49.0%)	10,967 (18.0%)	0.56 (0.54,0.57)	0.67 (0.65,0.69)
Officer in uniform	Y	2,287,516	1,138,109 (49.8%)	459,895 (20.1%)	0.38 (0.38,0.39)	0.66 (0.65,0.66)
	N*	907,788	632,323 (69.7%)	250,118 (27.6%)	1	1
Radio run	Y	726,152	351,623 (48.4%)	160,128 (22.1%)	0.85 (0.84,0.85)	1.07 (1.06,1.08)
	N*	2,469,152	1,418,809 (57.5%)	549,885 (22.3%)	1	1
Ongoing investigation	Y	424,648	268,718 (63.3%)	112,775 (26.6%)	1.06 (1.04,1.07)	1.15 (1.13,1.16)
	N*	2,770,656	1,501,714 (54.2%)	597,238 (21.6%)	1	1
Report by witness/victim	Y	363,836	223,462 (61.4%)	114,178 (31.4%)	1.53 (1.51,1.55)	1.66 (1.64,1.68)
	N*	2,831,469	1,546,970 (54.6%)	595,835 (21.0%)	1	1
Close to crime scene	Y	632,091	384,171 (60.8%)	164,041 (26.0%)	1.11 (1.10,1.12)	1.12 (1.11,1.13)
	N*	2,563,213	1,386,261 (54.1%)	545,972 (21.3%)	1	1
Sights/sounds of criminal activity	Y	76,023	39,921 (52.5%)	20,583 (27.1%)	0.98 (0.96,1.01)	1.34 (1.31,1.38)
	N*	3,119,281	1,730,511 (55.5%)	689,430 (22.1%)	1	1
Evasive response to questioning	Y	624,923	430,778 (68.9%)	182,639 (29.2%)	1.66 (1.64,1.67)	1.35 (1.34,1.36)
	N*	2,570,381	1,339,654 (52.1%)	527,374 (20.5%)	1	1
Associating with known criminals	Y	115,477	76,382 (66.1%)	33,269 (28.8%)	1.09 (1.06,1.11)	1.17 (1.15,1.19)
	N*	3,079,827	1,694,050 (55.0%)	676,744 (22.0%)	1	1
Carrying a suspicious object	Y	90,956	45,042 (49.5%)	21,993 (24.2%)	1.23 (1.20,1.26)	1.41 (1.38,1.44)
	N*	3,104,348	1,725,390 (55.6%)	688,020 (22.2%)	1	1
Fits a relevant description	Y	505,573	315,691 (62.4%)	144,332 (28.6%)	1.59 (1.57,1.61)	1.31 (1.29,1.32)
	N*	2,689,731	1,454,741 (54.1%)	565,681 (21.0%)	1	1
Casing a victim or location	Y	988,178	508,265 (51.4%)	188,514 (19.1%)	1.05 (1.04,1.06)	0.99 (0.98,1.00)
	N*	2,207,126	1,262,167 (57.2%)	521,499 (23.6%)	1	1
Acting as a lookout	Y	555,976	286,529 (51.5%)	107,898 (19.4%)	0.92 (0.91,0.93)	0.94 (0.93,0.95)
	N*	2,639,328	1,483,903 (56.2%)	602,115 (22.8%)	1	1
Wearing clothes commonly used in crime	Y	142,875	101,012 (70.7%)	40,267 (28.2%)	1.26 (1.24,1.29)	1.06 (1.04,1.08)
	N*	3,052,429	1,669,420 (54.7%)	669,746 (21.9%)	1	1
Actions indicative of a drug transaction	Y	284,498	140,992 (49.6%)	58,813 (20.7%)	1.24 (1.22,1.26)	1.20 (1.18,1.22)
	N*	2,910,806	1,629,440 (56.0%)	651,200 (22.4%)	1	1
Furtive movements	Y	1,510,560	1,006,551 (66.6%)	397,061 (26.3%)	1.71 (1.70,1.72)	1.30 (1.29,1.31)
	N*	1,684,744	763,881 (45.3%)	312,952 (18.6%)	1	1
Changed direction at sight of officer	Y	782,660	511,909 (65.4%)	210,583 (26.9%)	1.13 (1.12,1.14)	1.10 (1.09,1.11)
	N*	2,412,644	1,258,523 (52.2%)	499,430 (20.7%)	1	1
Actions of engaging in a violent crime	Y	275,167	204,802 (74.4%)	86,510 (31.4%)	1.73 (1.71,1.76)	1.36 (1.35,1.38)
	N*	2,920,137	1,565,630 (53.6%)	623,503 (21.4%)	1	1
Suspicious bulge	Y	294,839	269,267 (91.3%)	110,593 (37.5%)	4.00 (3.92,4.07)	1.58 (1.56,1.60)
	N*	2,900,465	1,501,165 (51.8%)	599,420 (20.7%)	1	1

Table E - Descriptive and inferential statistics relating covariates used in the model for the circumstances of and reasons for the stop to whether the suspect frisked/searched or had force used on them. The adjusted odds ratios (OR) are for the specific category of the covariate in the model with all the other covariates in Table S1 and the suspect's anthropometric values categorized by height and weight (Size). The values are given as N(% of sample) or OR(99% confidence interval), with the reference category denoted by *.

Factor	Term		Parameter Estimate	Standard Error	Test Statistic	p-value
Race	Omnibus		-	-	96.18	<0.0001
	Black		0.2153	0.0062	34.78	<0.0001
	Hispanic		0.1924	0.0063	30.63	<0.0001
	Other		-0.0219	0.0088	-2.48	0.0132
Height/ Weight Category	Omnibus		-	-	33.15	<0.0001
	H<66”, W<141 lbs.		-0.1061	0.0283	-3.75	0.0002
	H<66”, W∈ [141,205] lbs.		-0.0408	0.0205	-1.99	0.0465
	H<66”, W≥205 lbs.		0.0041	0.0767	0.05	0.9575
	H∈ [66",72"], W<141 lbs.		0.0655	0.0172	3.81	0.0001
	H∈ [66",72"], W≥205 lbs.		0.0440	0.0163	2.69	0.0071
	H≥73”, W<141 lbs.		0.0515	0.0801	0.64	0.5206
	H≥73”, W∈ [141,205] lbs.		0.0335	0.0147	2.28	0.0225
	H≥73”, W≥205 lbs.		0.0907	0.0228	3.97	<0.0001
Race-by-Height/ Weight Category Interaction	Omnibus		-	-	5.32	<0.0001
	Black	H<66”, W<141 lbs.	0.0673	0.0312	2.16	0.0310
		H<66”, W∈ [141,205] lbs.	-0.0191	0.0227	-0.84	0.4010
		H<66”, W≥205 lbs.	-0.0884	0.0849	-1.04	0.2978
		H∈ [66",72"], W<141 lbs.	-0.0386	0.0190	-2.03	0.0422
		H∈ [66",72"], W≥205 lbs.	-0.0038	0.0180	-0.21	0.8333
		H≥73”, W<141 lbs.	-0.0304	0.0874	-0.35	0.7277
		H≥73”, W∈ [141,205] lbs.	0.0447	0.0160	2.80	0.0051
		H≥73”, W≥205 lbs.	0.0112	0.0247	0.45	0.6509
	Hispanic	H<66”, W<141 lbs.	0.1410	0.0303	4.65	<0.0001
		H<66”, W∈ [141,205] lbs.	0.0264	0.0220	1.20	0.2297
		H<66”, W≥205 lbs.	0.0267	0.0849	0.31	0.7529
		H∈ [66",72"], W<141 lbs.	-0.0293	0.0197	-1.49	0.1367
		H∈ [66",72"], W≥205 lbs.	0.0282	0.0192	1.47	0.1409
		H≥73”, W<141 lbs.	-0.2176	0.1043	-2.09	0.0369
		H≥73”, W∈ [141,205] lbs.	-0.0093	0.0193	-0.48	0.6298
		H≥73”, W≥205 lbs.	-0.0141	0.0296	-0.48	0.6347
	Other	H<66”, W<141 lbs.	0.0151	0.0383	0.39	0.6935
		H<66”, W∈ [141,205] lbs.	-0.0660	0.0294	-2.24	0.0250
		H<66”, W≥205 lbs.	-0.1594	0.1315	-1.21	0.2255
		H∈ [66",72"], W<141 lbs.	-0.0740	0.0259	-2.86	0.0043
		H∈ [66",72"], W≥205 lbs.	0.0387	0.0314	1.23	0.2180
		H≥73”, W<141 lbs.	0.1462	0.1484	0.99	0.3243
		H≥73”, W∈ [141,205] lbs.	0.0781	0.0295	2.65	0.0081
		H≥73”, W≥205 lbs.	0.0418	0.0473	0.88	0.3771

Table F – The model estimates and statistical inference for race, height/weight category, and their interaction for the modeling of the odds of a suspect being frisked or searched. The omnibus tests are based on F-statistics with 3, 8, and 24 numerator degrees of freedom, respectively. There were approximately 3,200,000 degrees of freedom in the denominators of the omnibus tests and for the t-statistics for the individual parameters. Note that the parameters would need to be exponentiated to produce odds ratios.

Factor	Term		Parameter Estimate	Standard Error	Test Statistic	p-value
Race	Omnibus		-	-	29.93	<0.0001
	Black		0.0904	0.0072	12.62	<0.0001
	Hispanic		0.0937	0.0073	12.90	<0.0001
	Other		-0.0500	0.0103	-4.85	<0.0001
Height/ Weight Category	Omnibus		-	-	20.54	<0.0001
	H<66”, W<141 lbs.		-0.0328	0.0348	-0.94	0.3461
	H<66”, W∈ [141,205] lbs.		-0.0922	0.0256	-3.61	0.0003
	H<66”, W≥205 lbs.		-0.0125	0.0964	-0.13	0.8971
	H∈ [66",72"], W<141 lbs.		0.0118	0.0209	0.56	0.5739
	H∈ [66",72"], W≥205 lbs.		0.0894	0.0193	4.63	<0.0001
	H≥73”, W<141 lbs.		-0.0830	0.0992	-0.84	0.4031
	H≥73”, W∈ [141,205] lbs.		0.0447	0.0174	2.57	0.0102
	H≥73”, W≥205 lbs.		0.0717	0.0266	2.70	0.0069
Race-by-Height/ Weight Category Interaction	Omnibus		-	-	3.01	<0.0001
	Black	H<66”, W<141 lbs.	0.0153	0.0375	0.41	0.6825
		H<66”, W∈ [141,205] lbs.	0.0734	0.0276	2.66	0.0078
		H<66”, W≥205 lbs.	0.0060	0.1041	0.06	0.9540
		H∈ [66",72"], W<141 lbs.	-0.0025	0.0226	-0.11	0.9134
		H∈ [66",72"], W≥205 lbs.	-0.0122	0.0209	-0.58	0.5587
		H≥73”, W<141 lbs.	0.0516	0.1059	0.49	0.6260
		H≥73”, W∈ [141,205] lbs.	0.0117	0.0185	0.63	0.5267
		H≥73”, W≥205 lbs.	0.0252	0.0282	0.89	0.3719
	Hispanic	H<66”, W<141 lbs.	0.0768	0.0366	2.10	0.0358
		H<66”, W∈ [141,205] lbs.	0.1114	0.0269	4.15	<0.0001
		H<66”, W≥205 lbs.	0.0591	0.1037	0.57	0.5684
		H∈ [66",72"], W<141 lbs.	0.0146	0.0231	0.63	0.5272
		H∈ [66",72"], W≥205 lbs.	-0.0158	0.0219	-0.72	0.4695
		H≥73”, W<141 lbs.	0.1282	0.1206	1.06	0.2878
		H≥73”, W∈ [141,205] lbs.	-0.0194	0.0216	-0.90	0.3695
		H≥73”, W≥205 lbs.	0.0083	0.0324	0.26	0.7978
	Other	H<66”, W<141 lbs.	0.0202	0.0461	0.44	0.6618
		H<66”, W∈ [141,205] lbs.	-0.0091	0.0359	-0.25	0.7989
		H<66”, W≥205 lbs.	0.0546	0.1547	0.35	0.7243
		H∈ [66",72"], W<141 lbs.	0.0223	0.0307	0.73	0.4672
		H∈ [66",72"], W≥205 lbs.	-0.0570	0.0361	-1.58	0.1145
		H≥73”, W<141 lbs.	-0.1018	0.1836	-0.55	0.5793
		H≥73”, W∈ [141,205] lbs.	0.0678	0.0331	2.05	0.0404
		H≥73”, W≥205 lbs.	0.0344	0.0521	0.66	0.5088

Table G – The model estimates and statistical inference for race, height/weight category, and their interaction for the modeling of the odds of a suspect having force used on him. The omnibus tests are based on F-statistics with 3, 8, and 24 numerator degrees of freedom, respectively. There were approximately 3,200,000 degrees of freedom in the denominators of the omnibus tests and for the t-statistics for the individual parameters. Note that the parameters would need to be exponentiated to produce odds ratios.

Factor	Term		Parameter Estimate	Standard Error	Test Statistic	p-value
Race	Omnibus		-	-	177.76	<0.0001
	Black		0.2225	0.0072	30.73	<0.0001
	Hispanic		0.1831	0.0075	24.39	<0.0001
	Other		-0.0601	0.0103	-5.82	<0.0001
BMI Category	Omnibus		-	-	80.52	<0.0001
	Underweight		-0.0510	0.0412	-1.24	0.2163
	Overweight		-0.0803	0.0088	-9.13	<0.0001
	Obese		-0.0224	0.0146	-1.54	0.1235
Race-by-BMI Category Interaction	Omnibus		-	-	4.21	<0.0001
	Black	Underweight	-0.0237	0.0456	-0.52	0.6031
		Overweight	-0.0007	0.0097	-0.07	0.9450
		Obese	-0.0146	0.0160	-0.91	0.3629
	Hispanic	Underweight	-0.0289	0.0502	-0.57	0.5657
		Overweight	0.0260	0.0102	2.56	0.0104
		Obese	0.0077	0.0167	0.46	0.6450
	Other	Underweight	-0.0460	0.0647	-0.71	0.4774
		Overweight	0.0528	0.0147	3.60	0.0003
		Obese	0.0570	0.0259	2.20	0.0278

Table H – The model estimates and statistical inference for race, BMI category, and their interaction for the modeling of the odds of a suspect being frisked or searched. The omnibus tests are based on F-statistics with 3, 3, and 9 numerator degrees of freedom, respectively. There were approximately 3,200,000 degrees of freedom in the denominators of the omnibus tests and for the t-statistics for the individual parameters. Note that the parameters would need to be exponentiated to produce odds ratios.

Factor	Term		Parameter Estimate	Standard Error	Test Statistic	p-value
Race	Omnibus		-	-	39.56	<0.0001
	Black		0.0859	0.0083	10.29	<0.0001
	Hispanic		0.0817	0.0086	9.49	<0.0001
	Other		-0.0727	0.0120	-6.06	<0.0001
BMI Category	Omnibus		-	-	37.02	<0.0001
	Underweight		-0.1181	0.0515	-2.29	0.0219
	Overweight		-0.0666	0.0106	-6.3	<0.0001
	Obese		-0.0026	0.0175	-0.15	0.8825
Race-by-BMI Category Interaction	Omnibus		-	-	2.43	0.0094
	Black	Underweight	0.0684	0.0555	1.23	0.2181
		Overweight	0.0188	0.0114	1.66	0.0977
		Obese	0.0276	0.0188	1.47	0.1422
	Hispanic	Underweight	0.1123	0.0595	1.89	0.0589
		Overweight	0.0355	0.0118	3.01	0.0026
		Obese	0.0161	0.0194	0.83	0.4073
	Other	Underweight	0.1830	0.0774	2.37	0.0180
		Overweight	0.0347	0.0172	2.01	0.0442
		Obese	0.0187	0.0301	0.62	0.5350

Table I – The model estimates and statistical inference for race, BMI category, and their interaction for the modeling of the odds of a suspect having force used on him. The omnibus tests are based on F-statistics with 3, 3, and 9 numerator degrees of freedom, respectively. There were approximately 3,200,000 degrees of freedom in the denominators of the omnibus tests and for the t-statistics for the individual parameters. Note that the parameters would need to be exponentiated to produce odds ratios.

Figure Legends

Figure A – Predicted probabilities of the suspect being frisked or searched, for each height, weight, and race category, given the ‘average’ stop characteristics for a suspect with that race and size. The blue circle denotes white (W) suspects, the red diamond denotes black (B) suspects, and the green square denotes Hispanic (H) suspects. Error bars indicate the 99% confidence interval for the group estimates.

Figure B – Predicted probabilities of the suspect having force used on him, for each height, weight, and race category, given the ‘average’ stop characteristics for a suspect with that race and size. The blue circle denotes white (W) suspects, the red diamond denotes black (B) suspects, and the green square denotes Hispanic (H) suspects. Error bars indicate the 99% confidence interval for the group estimates.

Figure C – Predicted probabilities of the suspect having force used on him, for each BMI category-by-race group, given the ‘average’ stop characteristics for a suspect with that race and BMI classification. The blue circle denotes white suspects, the red diamond denotes black suspects, and the green square denotes Hispanic suspects. Error bars indicate the 99% confidence interval for the group estimates.

Figure A

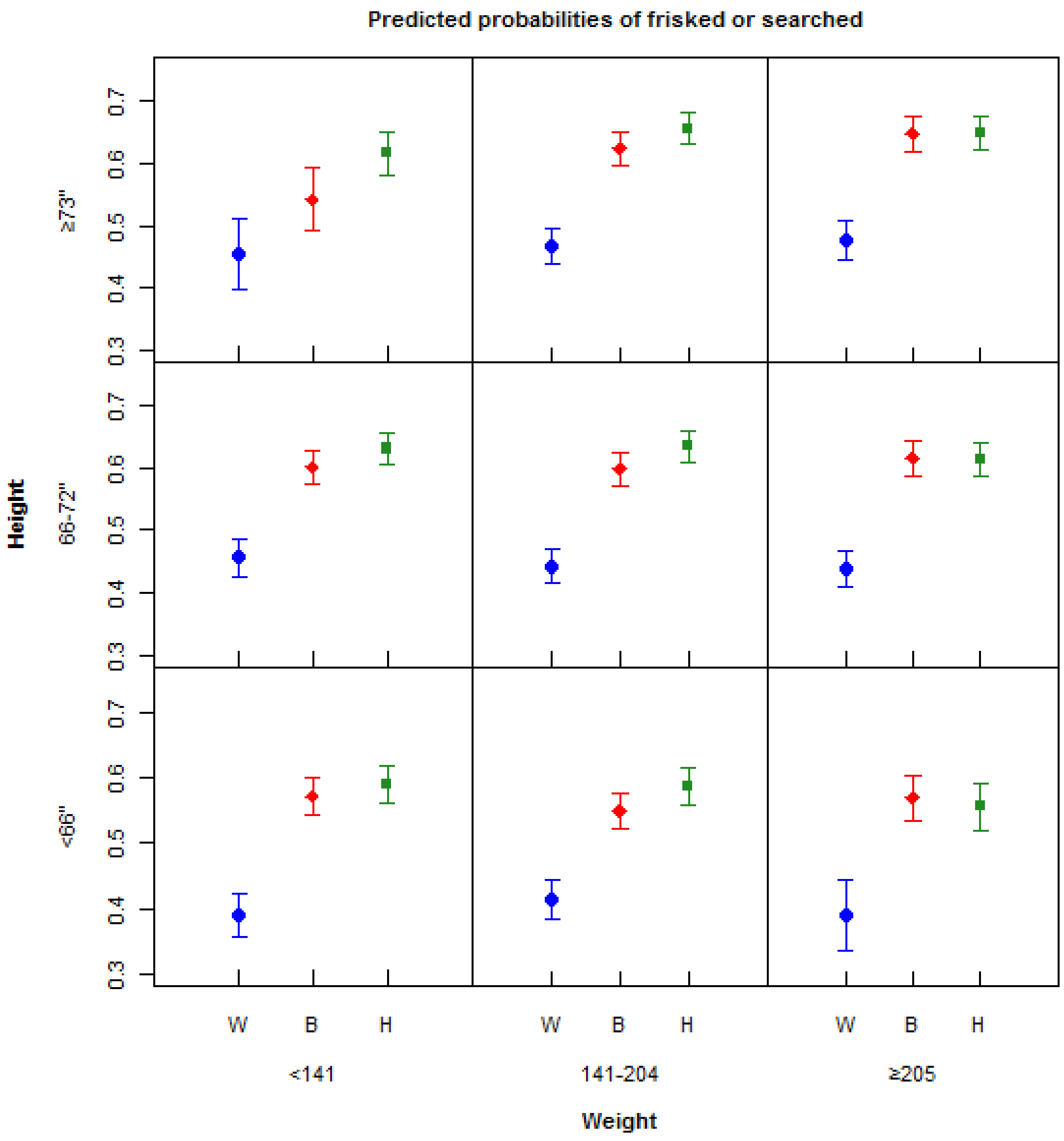
Predicted probabilities of frisked or searched
Height
≥73"
66-72"
<66"
0.3
0.4
0.5
0.6
0.7
W
B
H
<141
141-204
≥205
Weight

Figure B

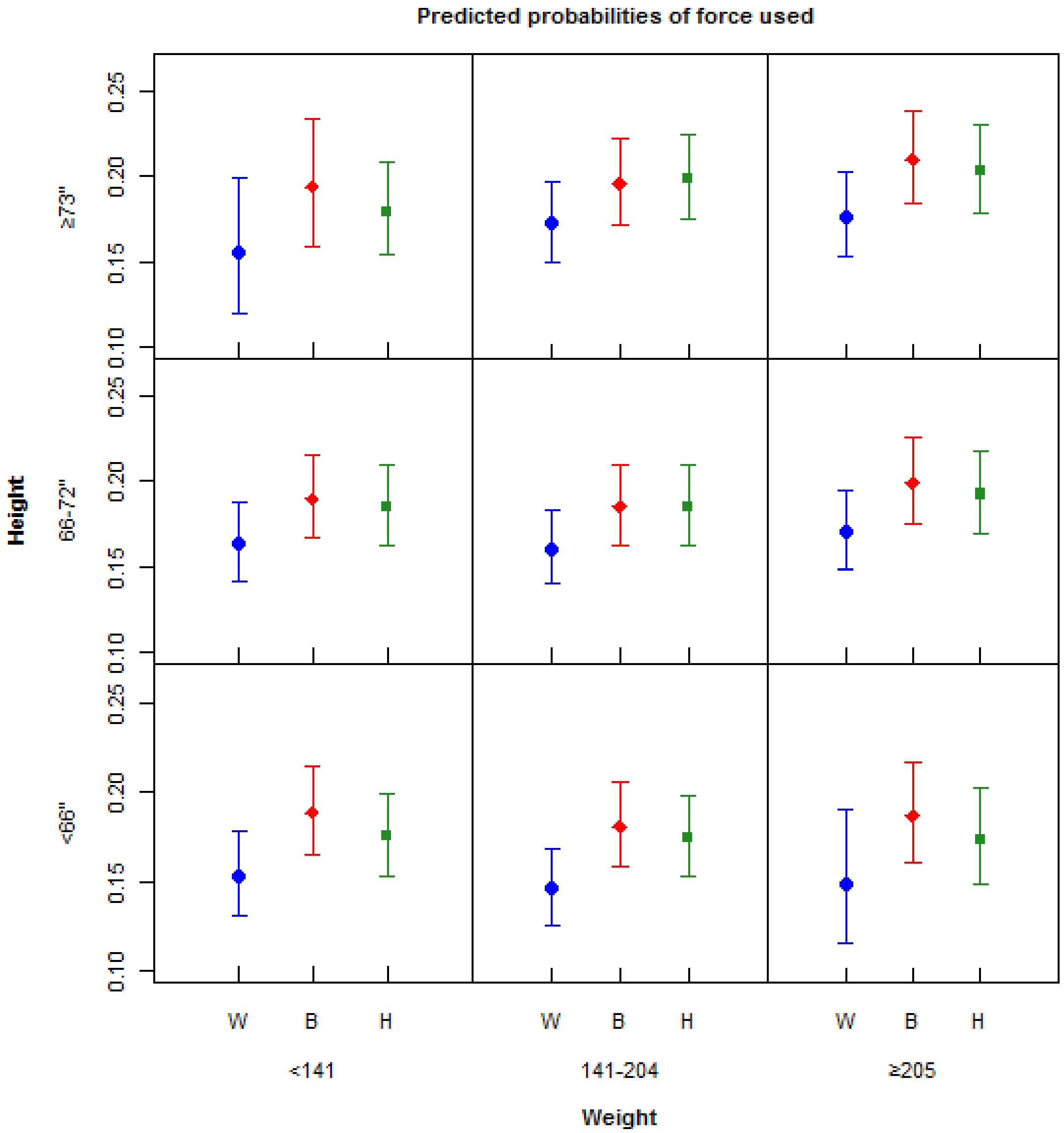
Predicted probabilities of force used
Height
≥73"
66-72"
<66"
0.10
0.15
0.20
0.25
W
B
H
<141
141-204
≥205
Weight

Figure C

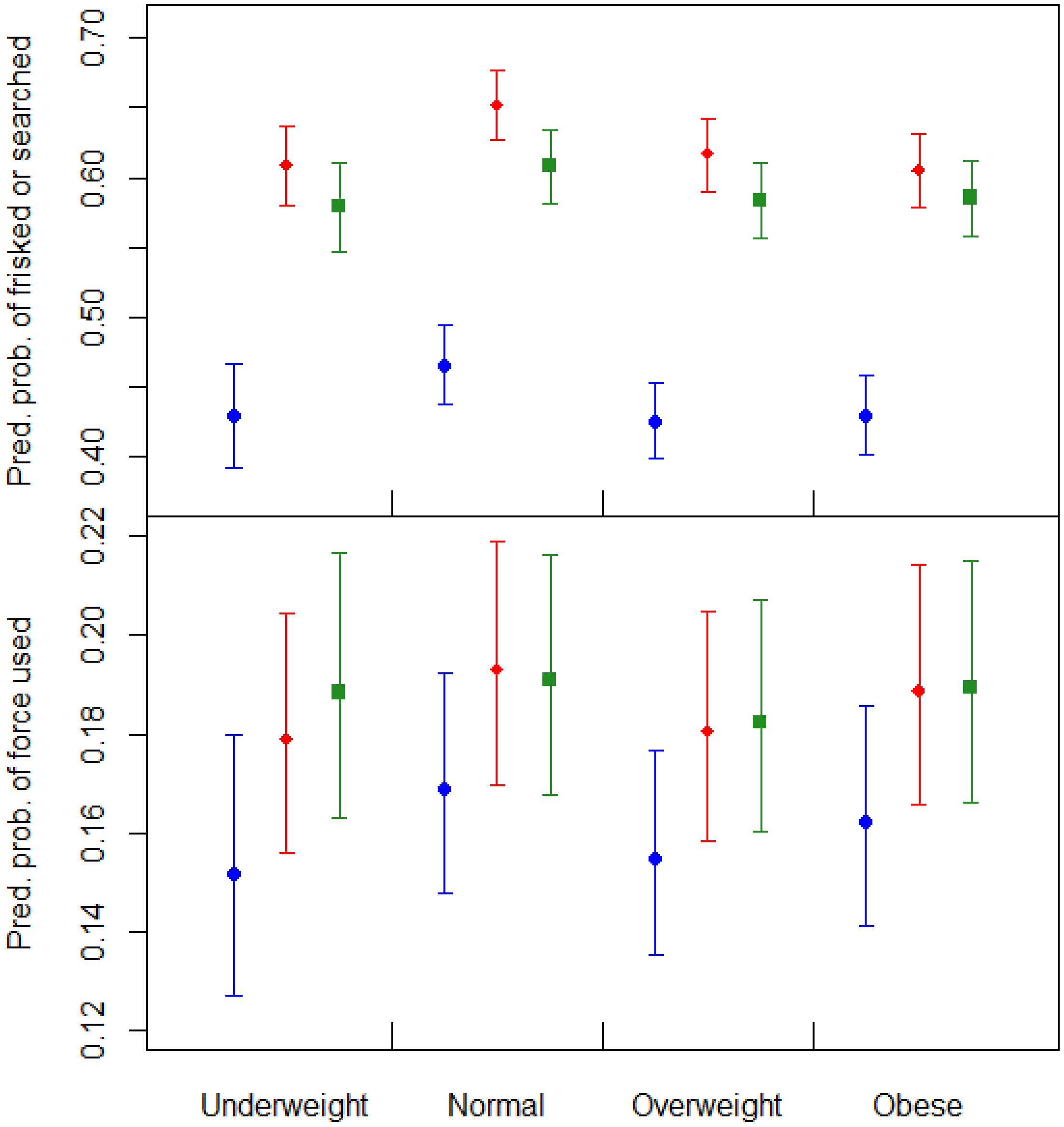
Pred. prob. of frisked or searched
0.40
0.50
0.60
0.70
Pred. prob. of force used
0.12
0.14
0.16
0.18
0.20
0.22
Underweight
Normal
Overweight
Obese
